# Complete Genome Sequence of the Urethral Catheter Isolate Myroides sp. A21

**DOI:** 10.1128/genomeA.00068-15

**Published:** 2015-03-05

**Authors:** Melanie Burghartz, Boyke Bunk, Cathrin Spröer, Sonja Voget, Rolf Daniel, Jörg Overmann, Martina Jahn

**Affiliations:** a Institute of Microbiology, Technical University Braunschweig, Braunschweig, Germany; b Leibniz Institute DSMZ-German Collection of Microorganisms and Cell, Cultures, Braunschweig, Germany; c German Centre of Infection Research (DZIF), Partner Site Hannover-Braunschweig, Braunschweig, Germany; d Department of Genomic and Applied Microbiology and Göttingen Genomics Laboratory, Georg-August-University, Göttingen, Germany

## Abstract

Myroides sp. A21, isolated from a urethral catheterized patient without symptoms of a urinary tract infection in Germany, proved to be extensively drug resistant. Here, we report the 4.16-Mb complete genome sequence of strain A21, carrying unusual pathogenicity islands and explaining the features of multidrug resistance.

## GENOME ANNOUNCEMENT

Myroides spp. have been reported to cause opportunistic infections (1). Though rare, fatal cases of tissue infections and nosocomial outbreaks have been described (2, 3). However, its pathogenicity is ill-defined and although infections caused by this bacillus are rare, it is notoriously resistant to multiple antibiotics.

For *de novo* genome assembly, sequence reads were generated by combination of single-molecule real-time (SMRT) and Illumina sequencing technologies. Sequencing was carried out on the PacBio RSII (Pacific Biosciences, Menlo Park, CA) using P4 Chemistry. Illumina libraries were run on an Illumina genome analyzer GAIIx, yielding 3.2 million 112 bp paired-end reads. Genome assembly was performed with the “RS_HGAP_Assembly.2” protocol included in SMRT Portal version 2.2.0, utilizing 496,985 postfiltered reads with an average read length of 6,019 bp. One contig was obtained, which was trimmed, circularized, and adjusted to *dnaA* (MYRA21_0001) as first gene. Quality improvement of the final consensus sequence was performed with Burrows-Wheeler Aligner (BWA) (4) mapping the Illumina reads onto the obtained contig. A final quality of QV60 was confirmed. Automated genome annotation was carried out using IMG (5). After automated prediction and subsequent manual curation, 3,650 protein-coding sequences (CDSs) and 136 RNA-coding genes were identified for the Myroides sp. A21 genome. Eight copies of the rRNA gene cluster were identified; three of them were resolved as a direct repeat of two rRNA gene clusters. There were 106 tRNAs and 6 noncoding RNAs (ncRNAs) predicted. The genome sequence was compared with M. odoratimimus CCUG 10230 and M. odoratus DSM 2801 using IMG’s “Genome Gene Best Homologs” function. Thereby, 293 unique CDSs were identified in the A21 genome. Five genomic islands were predicted by an IslandViewer analysis (6). One of them contains 52 predicted open reading frames. A surprising number of 10 gene regulators, including a ferric uptake regulator Fur homologue and a tetracycline responsive RteC regulator (7) were found. Genes for multidrug ABC transport system, an arsenical resistance protein and a chromate transporter contribute to the multiantibiotic and general resistance. The genes encoding hydrolases, a protein-tyrosine-phosphatase and an ADP-ribosylglycohydrolase might be involved in the attack and reprogramming of the host cell via protein modification (8). Additional potential ferrienterochelin and cholicin receptor and transport system further sustain the essential iron acquisition. Stress resistance might be mediated by a thioredoxin reductase (9). Mobility of this island might be endorsed by various transposase genes. A second mini island encodes 7 genes involved in LPS biosynthesis. Obviously, our Myroides isolate is well equipped for the infection of and long-term survival in the mammalian hosts.

### Nucleotide sequence accession number.

The genome sequence of the Myroides strain A21 is available in GenBank under accession no. CP010327.
